# Endobronchial and endoscopic ultrasound: it only takes an echobronchoscope to tango

**DOI:** 10.1002/rcr2.482

**Published:** 2019-08-27

**Authors:** Chun Ian Soo, Sze Shyang Kho, Boon Hau Ng, Siew Teck Tie

**Affiliations:** ^1^ Pulmonology Unit, Department of Internal Medicine National University of Malaysia (UKM) Medical Centre Kuala Lumpur Malaysia; ^2^ Division of Respiratory Medicine, Department of Internal Medicine Sarawak General Hospital, Ministry of Health Kuching Sarawak Malaysia

**Keywords:** Endobronchial ultrasound, endoscopic ultrasound echobronchoscope, lung cancer, mediastinal lymphadenopathy

## Abstract

Endobronchial ultrasound (EBUS) is indispensable when it comes to evaluation of mediastinal lesion and staging of lung cancer. The incorporation of endoscopic ultrasound using an echobronchoscope (EUS‐B) further extends this capability to the paraoesophageal and subdiaphragmatic structures. When combined, EBUS with EUS‐B increases the diagnostic yield. Both procedures can be done in a single session and by a single operator; this translates into an overall reduction in the healthcare cost.

## Introduction

Endobronchial ultrasound (EBUS) has been established as an important minimally invasive procedure for the investigation of mediastinal lesions and the staging of lung cancer. When combined with endoscopic ultrasound with bronchoscope‐guided fine‐needle aspiration (EUS‐B‐FNA), the technique allows for the augmentation of diagnostic potentials and increased precision in staging procedures.

## Case Series

We describe three cases of lung malignancy that employed three distinct applications of endobronchial ultrasound‐guided transbronchial needle aspiration (EBUS‐TBNA) and EUS‐B‐FNA. The first and second cases were performed at the National University of Malaysia (UKM) Medical Centre. The third case originated from Sarawak General Hospital.

### Case 1

**Figure 1:**
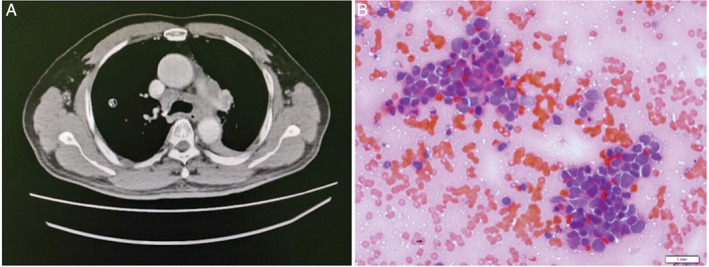
(A) Computed tomography thorax (axial view, soft tissue window) showed enlarged mediastinal lymph nodes at station (4R, 4L,5, 6 and 7). (B) Cellular smears obtained from station 4R lymph nodes composed of tightly packed malignant cells with nuclear moulding. The malignant cells have dispersed chromatin and scanty cytoplasm. Findings were consistent with the diagnosis of small cell carcinoma of the lung (haematoxylin and eosin stain, ×40‐fold magnification).

A 65‐year‐old man presented with a four‐week history of chronic cough and progressive dyspnoea. The computed tomography (CT) scan of his thorax revealed left lower lobe collapse with consolidation, presence of mediastinal lymphadenopathy (ML) and the absence of distant metastasis (Fig. 1). EBUS was performed under conscious sedation. Systematic assessment of the ML with linear EBUS showed enlarged nodes at stations 4R, 4L, 5, 6 and 7. Three aspiration passes were performed on station 4R of the ML. Following this, despite being given appropriate intravenous sedation (titrated to a maximum cumulative dose of 5 mg midazolam and 50 μg fentanyl), the patient had an intractable cough and reduced tolerance towards the procedure. This was overcome when EBUS was replaced with endoscopic ultrasound‐echobronchoscope (EUS‐B). Another three passes were performed on station 7 of the ML. Total procedure time was 42 min. A cytology examination was performed and revealed an abundant amount of abnormal cells on both of the samples obtained from stations 4R and 7. The patient was diagnosed with non‐resectable stage IIIB small cell lung carcinoma.

### Case 2

**Figure 2:**
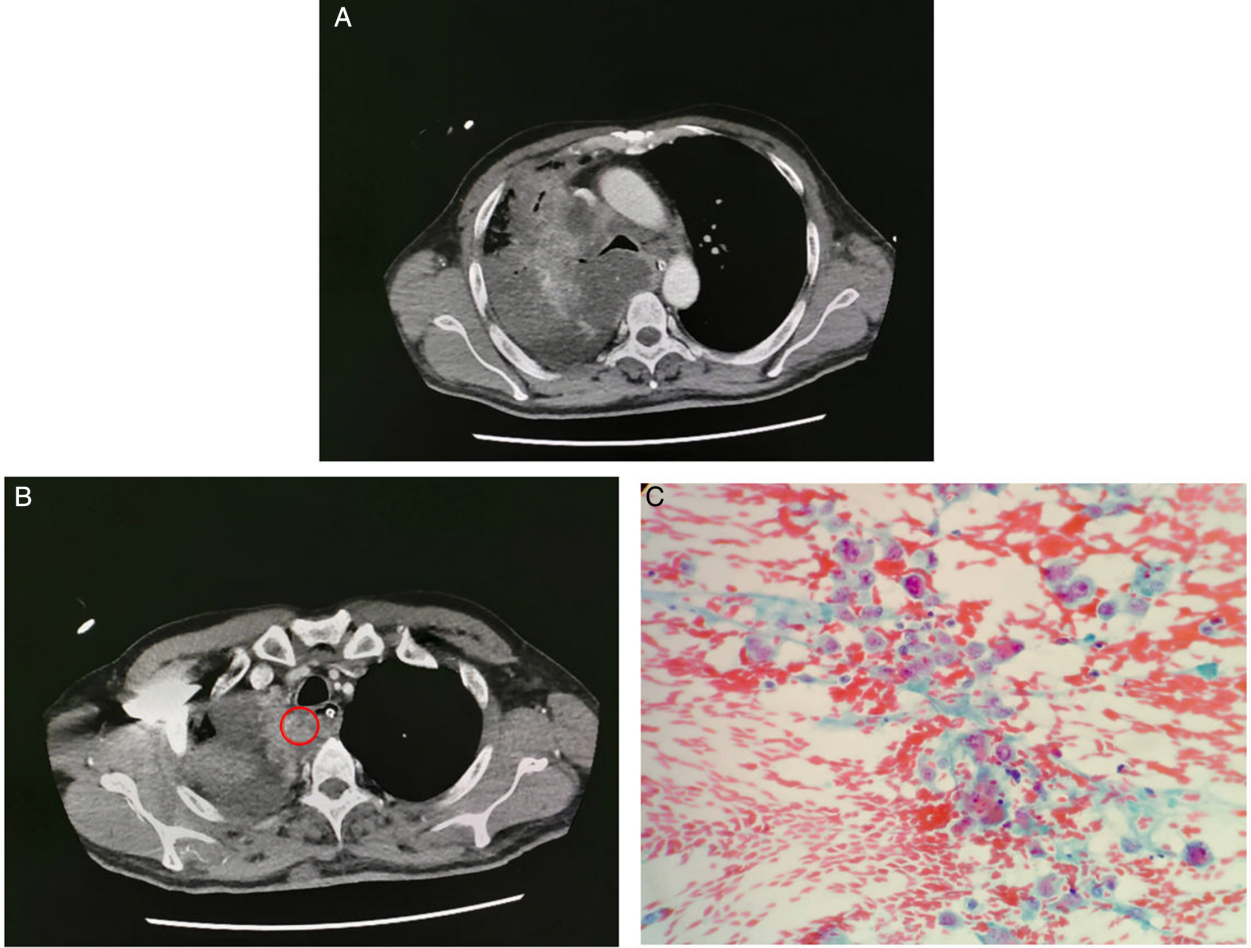
(A) Computed tomography thorax (axial view, soft tissue window, level of bifurcation of carina) showed a large heterogenous mass with multiple patchy necrotic areas on the right hemithorax. (B) Aspiration of the paraoesophageal non‐necrotic area which successfully clinched the diagnosis (red circle). (C) Cellular smear shows loose clusters and small sheets of malignant cells. These malignant cells display pleomorphism, hyperchromatic vesicular nuclei with prominent small nucleoli. Findings were suggestive of adenocarcinoma (Papanicolaou stain ×40‐fold magnification) (Video S1).

A 38‐year‐old man presented with a two‐month history of chronic cough. On arrival, he was intubated for severe acute respiratory failure with an arterial blood gas result of pH: 7.033, PaO_2_: 141 mmHg, partial pressure of carbon dioxide: 123 mmHg, and HCO^3−^: 21.3 mmol/L). A CT scan of the thorax was carried out to assess the suspicion of a widened mediastinum on the chest radiograph and confirmed the presence of a large, right‐sided heterogeneous pulmonary lesion with numerous ML. All lesions had multiple large, ill‐defined, scattered necrotic areas. We carried out a thorough review of the CT images to determine suitable non‐necrotic areas for aspiration (Fig. 2).

Bronchoscopy inspection revealed a narrowed central airway from the mid‐trachea extending down to proximal segments of the bilateral main bronchus. EBUS‐TBNA with three real‐time passes was performed at stations 4R and 7. This was followed by three FNA passes via EUS‐B on a non‐necrotic area adjacent to the proximal paraoesophageal area at the level of the sternal angle with the scope positioned at the 1 o'clock position. Total procedure time was 45 min. Final cytology results from EUS‐B guided aspirations confirmed the diagnosis of metastatic adenocarcinoma of the lungs.

### Case 3

**Figure 3:**
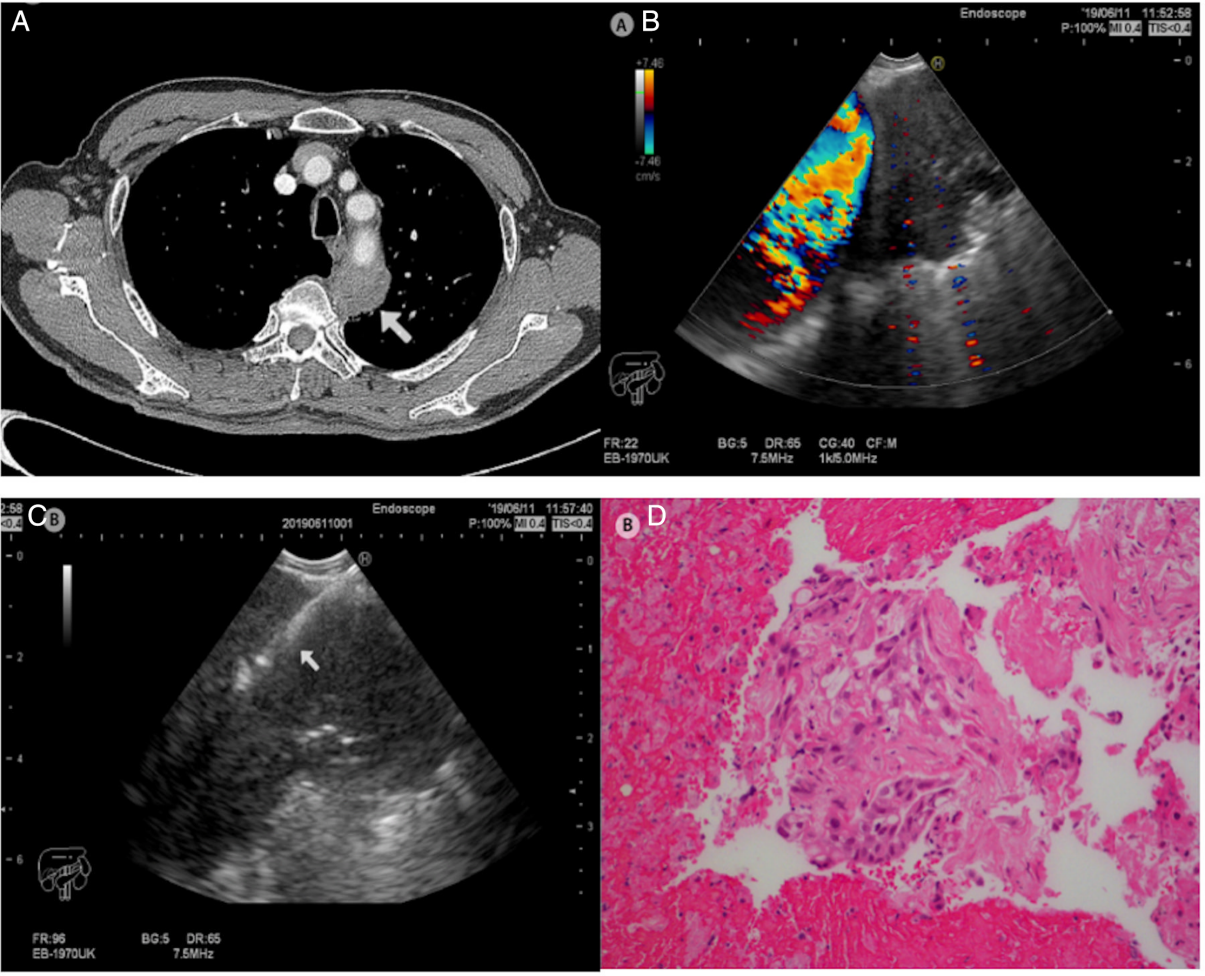
(A) Computed tomography thorax (axial view, soft tissue window). A heterogeneous mass at the apico‐posterior segment of the left upper lobe of the left lung abutting the posterior wall of the aortic arch (white arrow). (B) The mass adjacent to the descending aorta during endoscopic ultrasound using an echobronchoscope (EUS‐B) examination. (C) Real‐time endoscopic ultrasound with bronchoscope‐guided fine‐needle aspiration performed with 22G transbronchial needle aspiration into the mass (white arrow). (D) Clusters of pleomorphic malignant cells on histological examination of the tissue clot coagulum cell block (haematoxylin and eosin stain, ×20‐fold magnification).

A 70‐year‐old male was admitted for exacerbation of chronic obstructive pulmonary disease. A CT scan of his thorax was arranged to confirm the suspicion of a large bullae at the right lung. Results of the CT scan of the thorax revealed a heterogeneous lung mass at the posterior mediastinum that measured 3.4 cm × 3.1 cm (Fig. 3). The lesion demonstrated a standardized uptake value (SUV)max of 10.6 on 18F‐fluorodeoxyglucose positron emission tomography/CT (18F‐FDG PET/CT) scan with evidence of distant bone metastasis. Under conscious sedation, systematic assessment of the mediastinum using linear EBUS was insignificant. The heterogeneous mass was found to be paraoesophageal and adjacent to the descending aorta (Fig. 2A). A total of five real‐time passes (aspiration) were performed (Fig. 2B) with a procedure time of 35 min. Final cytology examination and immunohistochemical testing on aspiration samples was consistent with lung adenocarcinoma. Patient was diagnosed with stage IV disease.

## Discussion

The introduction of EBUS in the twenty‐first century propagated the idea of a minimally invasive approach for real‐time sonographic evaluations of benign or malignant mediastinal structures. Less than a decade later, the necessity to include the evaluation of structures in close proximity to the mediastinum required augmentation of the approach by introducing the EBUS through the oesophageal route. This led to the application of EUS‐B‐FNA.

EBUS has remained an indispensable tool for the evaluation of crucial mediastinal stations in lung cancer staging, especially low paratracheal (4R and 4L), subcarinal (7), hilar (10R and 10 L), and interlobar (11R and 11L). A combination of EUS‐B and EBUS improves in‐depth staging of lung cancer with better patient tolerance and access to evaluate upper paratracheal stations (2R and 2L), the left lower paratracheal station (4L) and possibly the left adrenal gland by a single operator during one session 1, 2, 3, 4. A recent study demonstrated that combined endosonography showed an increased sensitivity of 9% for N2/N3 disease in lung cancer staging compared to conventional PET‐CT‐targeted EBUS 5. An extension to the assessment of structures located on both sides of the diaphragm has also been described previously 4, 6.

The first case shows that EUS‐B compliments EBUS for lung cancer staging purposes and also serves as an alternative for challenging cases that require prolonged procedural time or a high amount of sedation. In contrast to the bronchus, endosonography through the oesophageal route causes significantly less coughing and oxygen desaturation. This improves patient tolerance and diagnostic yield, alleviating the concern of “tissue is the issue” and fear of inadequate samples. These results are even more apparent when dealing with patients with poor lung function.

Another advantage of EUS‐B is that it provides access for evaluation of mediastinal lesions adjacent to the oesophagus. Novel usage of EUS‐B for the diagnosis of centrally located intrapulmonary lesions has been described 7, 8. Two studies employing EUS‐B demonstrated a similar diagnostic yield of approximately 90% sensitivity and were comparable to conventional EUS for the diagnosis of intrapulmonary tumours; there were no reported complications 8, 9. A notable finding was that 45% of cases were diagnosed using only the transoesophageal approach with EUS‐B 8.

The second case demonstrated the practicality of EUS‐B when performed in a critically ill patient. As a result of the widespread areas of necrosis, endobronchial aspiration attempts at stations 7 and 4R only showed scanty material when examined by our cytotechnician. In addition, the narrowed left and right main bronchus and carina, which was distorted from external compression, prevented further assessment. When EUS‐B was employed in the area chosen prior to the procedure, the surrounding structures and necrotic areas of the mass were identified and avoided during aspiration. So, when EBUS‐TBNA fails, EUS‐B can provide a beneficial reduction of further unnecessary procedures and an overall reduction in the cost of treatment.

The third case depicts EUS‐B used in the evaluation and sampling of abnormal lesions which are not accessible by linear EBUS or radial EBUS. Utilization of EUS‐B in this case also averted unnecessary complications.

In the advent of EUS‐B, computed tomographic‐guided aspiration of centrally located lesions could possibly become a procedure of the past. Compared to its use with peripherally located lesions, CT‐guided aspiration of central lesions can be technically more challenging. In addition, assessment of central lesions by EUS‐B precludes potential complications, such as bleeding, iatrogenic injury to the surrounding organs, and, importantly, pneumothoraces attributed to radiologically guided sampling of centrally located mediastinal or pulmonary lesions 1, 7, 8. Invasive surgical staging can then be used as a reserved option for suspicious cases that failed endoscopic sampling.

With the growing evidence of the utility of EUS‐B, pulmonologists who are experienced in performing EBUS should explore and acquire the technical skills and competency needed to extend the diagnostic boundaries of echo‐endoscopes. A structured training programme could be the catalyst necessary to realize the full potential of both EBUS and EUS‐B.

### Disclosure Statement

Appropriate written informed consent was obtained from all three patients for the publication of this case series and the accompanying images.

## Supporting information


**Video S1.** EUS‐B procedure for case 2.
